# Editorial

**Published:** 1851-04

**Authors:** 


Dr. Taylor,—*Dear Sir:*—Permit me through the medium of the Dental Register, to announce the following discovery in the DENTAL ART which consists of a FUSIBLE CEMENT, by means of which porcelain teeth can be united to each other, and to metallic plates, so firmly as to preclude the possibility of being broken from a plate by use in mastication.

This cement when fused is harder and much stronger than the teeth, and cannot be acted upon by saliva or acids.

By this discovery five important points have been gained.

First,—Great strength. Second,—A most perfect artificial gum, as any desirable color can be given it, even the most delicate tints. Third,—It obviates the necessity of back-plating and soldering the teeth to the gum plate, thereby preventing any springing or warping of the plate upon which the teeth are set. Fourth,—The great facility with which teeth can be set. Fifth.—The cleanliness of the teeth when set, as not even the slightest moisture can get between the plate and teeth.

I have fully tested the practicability of this style of work in full and partial sets of teeth which are now being worn in this city, in which all the advantages above named are fully realized.*

Cincinnati, April 22, 1851. J. ALLEN, D.D.S.

*We have been shown some two or three sets of teeth mounted in the manner described by Dr. Allen, and if we mistake not, this improvement will entirely supersede what are called block teeth. If it continues to adhere as firmly to the plate and teeth as it appears to by inspection, we see no reason why it may not be made applicable to a large majority of plate jobs. It possesses all and more than all the advantages of block work, because you can select just such teeth as you desire, and arrange them as you like—then cement them to the plate, and it matters not if the teeth are ground to fit the plate or not; perhaps it is best they should not touch the plate, but permit the cement to flow under. The cases we examined were made on platinum and palladium plates. This we think necessary to avoid the springing of the the plate, for the heat requisite to flow the cement, would likely spring any gold plate, unless it was more than ordinarily pure. We know that Dr. Allen has been experimenting for several years, hoping to accomplish what he has now to appearance obtained, (time being necessary to test all such improvements), and we trust he may realize his most sanguine expectations, and make still more perfect that which he has thus far accomplished.—Ed.

### PORCELAIN TEETH.

We have been favored with a specimen of teeth made by Dr. Blandy, (Baltimore,) which are very natural in appearance. The body possesses a transparency very desirable, and appears to be made of materials thoroughly prepared, and the coloring for a light shade is very fine. We understand the Dr. is about entering very largely into the manufacture of teeth.

---

### STOCKTON'S TEETH.

We were shown a day or two since new samples of the above, the palital face of which are made so as to receive the gold backings. We think an operation finished up with these teeth would present a fine appearance. We regard, however, the form of the teeth on their labial face as a more important improvement, and we are glad to say that these approach nearer the natural organs in this respect than any we have seen from this establishment heretofore.

What has become of the fine gum teeth received from this manufactory some two or three years since? The lot sent to this market was used up in two or three months. The gum has sinse grown rather sickly, and needs, as we think, a dose of California.

We are glad to see signs of an awakening in this camp.

---

### ALCOCK'S TEETH.

These teeth have been in our market for a year past with very little effort to make them known; and yet the assortment was very soon so much culled out that it was difficult to make a good selection. We hope this will not be the case hereafter. Many of our dentists regard them as the most natural in form, and the strongest teeth made.

---

### MORTON'S TEETH.

Dr. Edwards has quite an assortment of these teeth. They possess in form a very natural appearance, and their adaptation for full sets (particularly where large gum teeth are required) answer a fine purpose. The coloring of the teeth and gum, however, lack a life-like appearance. We have, however, inserted but one set of these teeth. They stood well the heat of soldering, and look better in the mouth than we supposed.

---

### JONES, WHITE & CO'S TEETH.

These teeth are well formed on their cutting surface, and hence can be admirably articulated without marring their beauty by grinding. The gum of these we regard as very superior, and the pearl and yellow shades as fine as any we may soon expect. We are not yet satisfied that the teeth are as strong as some made by Stockton or Alcock.

### DENHARD'S TEETH.

These teeth are manufactured in Cincinnati, and by many are very much liked. The difficulty in getting proper moulds was for a long time a serious difficulty. This has to a great extent been overcome, and the teeth are better shaped than heretofore. Some of the dark shades are very natural, and this establishment in our city is of essential service to the profession in this section, furnishing to order such teeth as are often needed for anomalous cases and which cannot be had at any of our depots. Mr. Denhard, for his unwearied exertions to supply the profession in such cases, deserves their warmest gratitude.

---

### TO THE MANUFACTURERS OF PORCELAIN TEETH.

*Gentlemen,*—We are glad we have it in our power to say somewhat in commendation of all—and for your unwearied exertions to perfect this important manufactory, accept our sincere thanks. When we look back twenty or even ten years, we see that much—very much has been accomplished. We still keep some of the old stock for comparison. But permit us, in the close of this article, to throw out a few hints, which if taken kindly, may be of service to you all as well as to the profession. First, take time to prepare the material well. We may be wrong but we think that the feldspar, silex, &c., cannot be too finely pulverized. We notice some teeth which look as if they had been moulded of an impalpable powder. They are hard and grind smooth and if ground to the lower pin will not scale out and injure the teeth.

Second; have your coloring matter of such a quality and so thoroughly infused into the body of the teeth that it may not be affected by the heat of soldering. Third, the teeth should be all made for a special class in the mouth, for instance, an inferior tooth is never a superior one. True, we see that some dentists select without any regard to this rule, and hence instead of restoring the lost expression they merely change it. The first bicuspids fit up better if made a little smaller than the second, at least not so thick. We never can see the propriety of placing the platina pins cross-ways of the long teeth and the reverse in the short ones, and it is important that the bicuspids and molars below should be so made that the palital border of the tooth may rest on the plate. Many of us would like to see the second bicuspids above made with a hole to receive a head for the spring, and when thus made, so formed in size, length, &c., as to suit the other teeth of the same manufactory—we don't like to mix your teeth, gentlemen in the same operation.

Fourth; Let the platina pins be large and long enough to answer two purposes—first so large that we may have the necessary strength, and second, so long that when one of your teeth breaks off we may be able to rivet another on.

Fifth; Please recollect that the gum teeth are being used more and more every year, and that the gum should be strong and never fade in the mouth.

We have always been an advocate for the natural formation of the tooth. Nature's work we try to imitate, and when we get the form of teeth and shade of color to correspond with those in the mouth and have sufficient strength, we are content.

We find, gentlemen, that there are quite a number of you engaged in this business, and we expect to be benefitted thereby, for "competition is the life trade." Bear in mind, gentlemen and we hope the profession will also, bear in mind that it is far more important to increase the quality of these precious gems than reduce the pricc. Do not labor to glut the market but to excel.

### OHIO COLLEGE OF DENTAL SURGERY.

It will be seen by the announcement of this Institution that a change in the Faculty to some extent has taken place. This was rendered necessary, first, by the resignation of Dr. Leslie last winter, when the course was about half through; and second, because of the purchase of the Institution by various members of the Profession and the nomination by them of Dr. Allen to this chair. Dr. Allen has been for more than twenty years a practitioner of the profession in this city, and is especially devoted to Mechanical Dentistry.

Dr. Van Emon, a graduate of the school, and for eight or ten years a practitioner, takes the place of Dr. King, as Demonstrator, who has recently settled in Pittsburgh.

Dr. Van Emon will in addition to his duties as Demonstrator give a series of lectures on Dental Chemistry.

The prospects for the school are at this time more flattering than at any previous period, and a much larger class is expected than at any previous session. The profession will now have a permanent place for the meeting of their association, and we hope soon to see an Anatomical and Dental Museum and Library which will be of credit to the profession. And it is intended hereafter to keep the Dental Infirmary open all the year, so that students may at all times have an opportunity for acquiring practical knowledge. There are yet some five or six shares of stock which we hope some of the profession will subscribe for. There are thirty-eight shares in all, each share being $100, payable as follows; $40 first of August, 1851; $30 first of August, 1852; and $30 first of August, 1853. The stockholders get from the faculty 6 per cent. clear of all insurance, repairs, taxes, &c., &c., on the property.

The object is to have the stock so distributed that each stockholder will ultimately have but one share, and if necessary divide the shares and increase the number of stockholders. The Institution we wish to be in the hands of the profession, controlled and directed by them through the trustees, appointed by the Legislature. The Institution has a good and liberal charter, and is managed by an intelligent and able Board of trustees.

---

### BOND'S DENTAL MEDICINE.

The above is the title of a practical treatise on Dental Medicine, just from the press of Lindsay and Blakiston, Phil. And from the pen of Thomas E. Bond, A.M., M.D., Professor of Special Pathology and Therapeutics in the Baltimore College of Dental Surgery. Practicing physicians should read it and become more familiar with the effect of dental disease on the general system. Dentists should read it and learn that their profession is something more than a mechanic art. The work is well suited to the student of both medicine and dentistry, and we are glad to see such works published.

---

### ETHER AND CHLOROFORM IN SURGERY, DENTISTRY, &c.

This is a work also just published by Lindsay and Blakiston, and written by J. F. B. Flagg, M.D., Surgeon Dentist, Philadelphia. It gives a history of Ether and Chloroform. The mode of preparing, administering, &c., &c. Also the diseases to which it is applicable and the result of many interesting cases. We have not as yet had time for a careful perusal of this work containing one hundred and eighty pages, but have read enough to find much good practical matter, and which coincides with our own observation. The work is equally interesting to the medical as well as dental practitioner.

### MEDICAL STUDENTS' GUIDE IN EXTRACTING TEETH.

This is a small work of some seventy-five pages also from the press of Lindsay and Blakiston, by S. S. Horner, practical dentist.

This work is especially intended for the medical student, and gives some general directions for the use of the Key, Forceps, Punch, Elevator, Screw, &c., &c. Many of our physicians are obliged to extract teeth, and such should avail themselves of every possible means which may facilitate this much dreaded operation; to such this work would be of service.

The above works are for sale by Dr. J. M. Brown, Dental Depot, Cin.

---

Our correspondent A. F. E. is informed that his article is of too personal a character to be inserted in the Register. The gold sponge is, we believe, manufactured by Dr. Maire of New York, and has been experimented with by some of our eastern dentists, but we know not with what success, but we hope with better than that made on the teeth of A. F. E.

---

### ANOMALOUS CASES.

We have recently received two or three specimens of this character, which are worthy of notice and will be referred to in our next.

---

### ODONTALGIA.

We are requested by Dr. Robinson to state that in his work, when speaking of the subdivision of Odontalgia, he inadvertently omitted giving Dr. S. P. Hullihen of Wheeling, Va., the credit due to him, as the first to publish the views therein set forth upon this subject.—*American Journal of Dental Science.*

We are glad to see this correction, and take pleasure in placing Dr. Robinson aright before the profession.

---

The Mississippi Valley Association, it will be recollected, meets in Louisville, Kentucky, on the second Wednesday of September next.

---

An article on Salivary Calculus is crowded out of this Number for want of room.

